# Case Report and Literature Review: Bisphosphonate, Sirolimus, and Atenolol Treatment in a 4-Year-Old Child Diagnosed with Gorham–Stout Disease

**DOI:** 10.3390/ph16101504

**Published:** 2023-10-23

**Authors:** Su Jin Park, Jae Won Yoo, Moon Bae Ahn

**Affiliations:** 1 Divison of Endocrinology, Department of Pediatrics, Seoul St. Mary’s Hospital, College of Medicine, The Catholic University of Korea, Seoul 06591, Republic of Korea; jetaime_7@naver.com; 2 Division of Hematology and Oncology, Department of Pediatrics, Seoul St. Mary’s Hospital, College of Medicine, The Catholic University of Korea, Seoul 06591, Republic of Korea; hoiring0209@gmail.com

**Keywords:** Gorham–Stout disease, vanishing bone disease, bisphosphonate, sirolimus, atenolol

## Abstract

We report a 4-year-old with Gorham–Stout disease (GSD) who was treated with a combination of bisphosphonate, sirolimus, and atenolol. A previously healthy 4-year-old girl presented with back pain after falling on her back 2 months prior. Thoracolumbar spine X-ray revealed diffuse compression spinal fractures in T9-L2. Magnetic resonance imaging (MRI) confirmed multiple compression fractures at T9-L5 and revealed a paraspinal mass along the T1-L1 level. Based on clinical, radiological, and histopathological findings, Gorham–Stout disease was diagnosed. Treatment with sirolimus (0.5 mg twice daily, 1.6 mg/m^2^) was initiated and intravenous bisphosphonate (pamidronate, 1 mg/kg for 3 days, total 3 mg/kg every 4 months) was added for back pain; she had immediate improvement in back pain. After 9 months with this treatment, she had a mild increase in paraspinal lymphangiomatosis and aggravation in T9-L5 compression fractures; atenolol was administered. The patient underwent 11 months of combination treatment with bisphosphonate, sirolimus, and atenolol, and MRI showed mild degree of reduction in the paraspinal lesions at L1-L5. The patient is currently in stable condition with no back pain or side effects. The triple combination treatment with bisphosphonate, sirolimus, and atenolol may be helpful in stabilizing the disease course of GSD.

## 1. Introduction

Gorham–Stout disease (GSD), also known as vanishing bone disease, is an extremely rare skeletal disorder characterized by the proliferation of blood vessels and lymphatic channels within bone, resulting in progressive osteolysis [1]. GSD can affect any part of the skeleton, axial or appendicular, although the upper part of the body including the maxillofacial bones, clavicles, vertebrae, ribs, and the pelvic girdle are the most frequently involved [2,3]. Clinical manifestations of GSD include local pain and swelling in the affected areas, progressive bone deformities, functional impairment, and muscle weakness [4]. Patients may also be asymptomatic until spontaneous or traumatic fracture occurs. Pleural effusion or chylothorax may occur due to the extension of lymphoangiogenic invasion into the pleural cavity or thoracic duct, leading to poor prognosis [5].

GSD is diagnosed based on clinical, radiological, and histological features. However, because there are no specific biomarkers or radiological or histological findings that definitively diagnose GSD, its diagnosis is often challenging. It is usually diagnosed after the exclusion of other infectious, inflammatory, endocrine, and neoplastic diseases that may involve massive osteolysis. Although there is no standardized algorithm for diagnosis, Heffez et al. suggested eight diagnostic criteria for GSD: (1) positive biopsy for angiomatous tissue; (2) absence of cellular atypia; (3) minimal or no osteoblastic reaction and absence of dystrophic calcifications; (4) evidence of local, progressive bone resorption; (5) non-ulcerative lesion; (6) absence of visceral involvement; (7) osteolytic radiographic pattern; and (8) negative hereditary, metabolic, neoplastic, immunologic, or infectious etiology [6].

Since its first definition by Gorham and Stout in 1955 [1], approximately 350 cases of GSD have been reported [3]. Owing to the rarity of the disease, the pathogenetic mechanisms causing proliferation of angiomatous structures and osteolysis remain poorly understood. As a result, there are no established treatment protocols. Treatment options such as pharmacological therapy, radiotherapy, and surgical resection have been proposed. Bisphosphonates, interferon alfa-2b, calcium, and vitamin D supplements are the most commonly used monotherapies or combinations thereof [3,4]. Other drugs such as bevacizumab, denosumab, calcitonin and, recently, with encouraging results, sirolimus, have been used [7,8,9]. 

Herein, we report a case of a 4-year-old with GSD who was treated with a combination of bisphosphonate, sirolimus, and atenolol.

## 2. Case Report

A previously healthy 4-year-old girl presented with back pain after falling on her back, which gradually increased over the previous 2 months. Physical examination revealed no erythema or swelling on the back but mild midline spine tenderness. Serum calcium, phosphorus, intact parathyroid hormone, and alkaline phosphatase were within normal range, whereas serum 25-hydroxyvitamin D was slightly low (16.34 ng/mL, reference range 20–100 ng/mL). Notably, bone-specific alkaline phosphatase, a useful marker for bone formation, was slightly low (24.9 μg/L, reference range 32.8–105.5 μg/L), and carboxyterminal telopeptide of type I collagen, a marker for bone resorption, was normal (1.30 ng/mL, reference range 0.63–1.80 ng/mL), considering the sex- and age-specific reference intervals [10].

**Figure 1:**
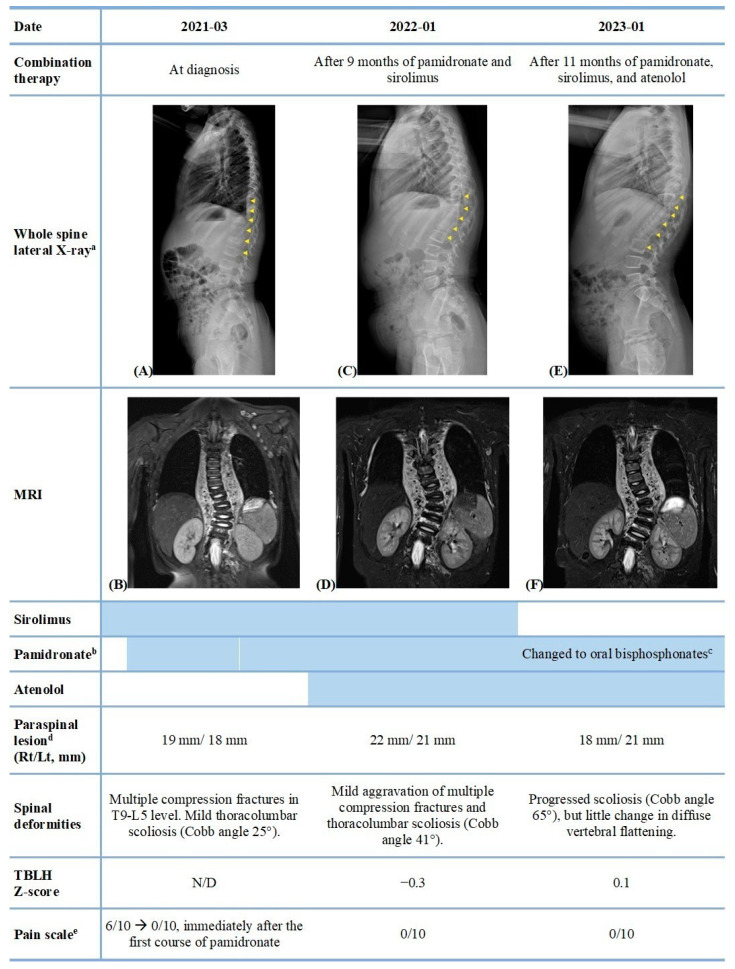
Clinical and radiological response to the combination therapy with pamidronate, sirolimus, and atenolol. (**A**) Thoracolumbar spine X-ray at diagnosis revealed diffuse spinal compression fractures in T9-L2 rather than a focal fracture caused by a back injury (**B**) T2-weighted coronal plane sequence at diagnosis confirmed multiple compression fractures at T9-L5 level, and revealed a paraspinal mass along the T1-L1 level, directly adjacent to the affected bones. (**C**) Thoracolumbar spine X-ray after 9 months of pamidronate and sirolimus treatment showed moderate thoracolumbar scoliosis and ongoing bone resorption in the affected spinal areas. (**D**) Whole-body MRI performed 9 months after pamidronate and sirolimus treatment showed a mildly increased extent of paraspinal soft tissue lesions and mild aggravation of multiple compression fractures at the T9-L5 level. (**E**) Thoracolumbar spine X-ray after 11 months of pamidronate, sirolimus, and atenolol treatment showed little change in diffuse vertebral fracture, although there was a progression of scoliosis. (**F**) The most recent MRI revealed further regression of the paraspinal soft tissue involvement at the L1-L5 level. Abbreviations: MRI, magnetic resonance imaging; Rt, right; Lt, left; TBLH, total body less head; N/D, Not done. (a) The yellow arrows indicate vertebral fractures shown in X-rays. (b) Throughout bisphosphonate treatment, cholecalciferol 1000 IU per day was administered orally. (c) Alendronate 5mg daily. (d) The largest lesion dimensions were measured. (e) Assessed with the Wong–Baker FACES pain rating scale.

Thoracolumbar spine X-ray revealed diffuse spinal compression fractures in T9-L2 rather than a focal fracture caused by a back injury (Figure 1A). Considering the low-impact injury to the back, the observed diffuse vertebral flattening was inconsistent with the injury context. Mild thoracolumbar scoliosis was also observed (Cobb angle 25°). The T2-weighted magnetic resonance imaging (MRI) not only confirmed multiple compression fractures at T9-L5 level, but also revealed a paraspinal mass along the T1-L1 level, directly adjacent to the affected bones (Figure 1B). The paraspinal mass showed T2-high, T1-low signal intensity with homogenous enhancement, which seemed similar to the vascular structures. Soft tissue masses with similar characteristics were observed in the anterior superior mediastinum, the left supraclavicular region, and the right lateral abdominal wall. To further confirm the diagnosis, soft tissue and bone biopsies were obtained from the supraspinous area of the T12 vertebrae. During open biopsy, serosanguinous fluid flowed out from the soft tissue attached to the T12 vertebrae. Soft tissue biopsy revealed that the soft tissue lesion was composed of numerous thin-walled vascular channels and fibrous tissues, similar to hemangiomas. Bone biopsy showed focal fibrosis. No inflammatory cell infiltration or cell atypia was observed in the soft tissue, bone, or serosanguinous fluid. Neoplastic process was ruled out. Overall, the histopathological findings of nonspecific vascular proliferation of the soft tissue mass directly adjacent to the affected bones, in correlation with the imaging, suggested skeletal angiomatosis with extensive manifestations in the spinal vertebrae. Based on the clinical, radiological, and histopathological findings, the patient was diagnosed with GSD.

Considering her young age and the fact that she showed no neurological deficits thus far, we preferred to control disease progression with pharmacologic therapy first rather than radiotherapy or surgical resection. Treatment with sirolimus (0.5 mg twice daily, 1.6 mg/m^2^), the mammalian target of the rapamycin (mTOR) inhibitor, which has anti-tumor and anti-angiogenic effects, was initiated. After 1 month of sirolimus treatment, the patient consistently complained of local back pain. Intravenous bisphosphonate treatment was started (pamidronate, 1 mg/kg for 3 consecutive days, total 3 mg/kg every 4 months). Vitamin D3 (cholecalciferol 1000 IU per day) was added throughout the bisphosphonate treatment to optimize the serum vitamin D level [11]. Following combination treatment with sirolimus and bisphosphonate, the patient had immediate improvement in back pain. She experienced pain relief shortly after the first course of intravenous pamidronate, as her self-reported degree of pain decreased to a score of 0/10 from the pretreatment score of 6/10 using the Wong–Baker FACES pain rating scale [12]. Furthermore, her parents reported that she complained of pain less often, even after low-impact falls, and with less intensity. Trough level of sirolimus was regularly monitored, targeting the range between 5 and 15 ng/mL, and the dose was escalated from 1 mg (0.5 mg twice a day) to 2.75 mg (1.5 mg in the morning, 1.25 mg in the evening) daily after 2 months; the dosage was maintained for 9 months. At this point, the patient was 5 years old, and her initial dual-energy X-ray absorptiometry was performed. Her age-matched total body less head Z-score was −0.3. In addition to the combination treatment, brace application was recommended for the patient; however, the brace had not been worn as prescribed due to discomfort. Thoracolumbar spine radiography revealed moderate thoracolumbar scoliosis (Cobb angle 41°) and ongoing bone resorption in the affected spinal areas (Figure 1C). Whole-body MRI performed 9 months after treatment with pamidronate and sirolimus revealed a mildly increased extent of paraspinal soft tissue lesions and mild aggravation of multiple compression fractures at the T9-L5 level (Figure 1D). Atenolol, a selective β_1_-blocker, was added (1.5 mg/kg/day) to inhibit proliferation of vascular and lymphatic channels. There have been a few reports regarding the beneficial effect of propranolol, a non-selective β-blocker, in the treatment of GSD due to its antiangiogenic activity [13,14,15]. Atenolol was chosen in this case based on recent studies supporting its efficacy and safety over propranolol for infantile hemangiomas [16,17]. Four months after the triple combination therapy with pamidronate, sirolimus, and atenolol, a follow-up whole-body MRI was performed. The follow-up MRI indicated a decreased extent of the paraspinal soft tissue lesion at the L1-L5 level and little change in spinal compression fractures of the T9-L5 level. As the lesions improved, the patient underwent an additional 7 months of triple combination therapy, with a total treatment duration of 11 months. The most recent MRI revealed further regression of the paraspinal soft tissue involvement at the L1-L5 level and a slight regression of the cutaneous involvement at the right lateral abdominal wall (Figure 1F). Follow-up dual-energy X-ray absorptiometry showed an increased total body less head Z-score of 0.1 compared with the previous data of −0.3. Thoracolumbar spine radiography showed little change in diffuse vertebral fracture, although there was a progression of scoliosis (Cobb angle, 65°) (Figure 1E). The clinical and radiological responses to combination therapy are summarized in Figure 1.

Throughout triple combination therapy with pamidronate, sirolimus, and atenolol, the patient experienced one episode of mild fever during the third course of pamidronate and two episodes of self-limiting oral mucositis and mild thrombocytopenia. Low-grade fever occurred, up to 37.8 °C, during the third course of pamidronate infusion, after the second dose of pamidronate administered. The fever subsided in a few hours without antipyretics. Oral mucositis and mild thrombocytopenia were considered side effects of sirolimus. Two episodes of oral mucositis occurred while on a year of sirolimus treatment and both episodes resolved in a few days. Mild thrombocytopenia was first identified after 5 months of sirolimus treatment, and a platelet count between 100 and 150 × 10^9^/L was sustained for a year. During the second year of sirolimus treatment, further decrease in platelet count to 80–100 × 10^9^/L was noted. The patient showed no bleeding symptoms, and no dose reduction was applied. However, since the recent radiological evaluation, her disease status has been considered stable; sirolimus was discontinued, and intravenous pamidronate was switched to oral alendronate (5 mg daily). Hence, she is currently receiving a combination treatment of oral bisphosphonate and atenolol. Her platelet count has shown partial response to suspension of sirolimus, ranging from 100 to 125 × 10^9^/L. The patient is in stable condition with no back pain or treatment-related adverse events. 

## 3. Discussion

GSD is characterized by angiogenesis, lymphangiogenesis, and massive bone resorption without new bone formation [1,6]. Increased osteoclastic activity, proliferation of blood and lymphatic channels, and lack of osteoblast function are three essential features of GSD. Considering the complex pathogenetic mechanisms of this disease, Rossi et al. [18] proposed a multi-targeted approach combining anti-osteoclast drugs, angiogenesis inhibitors, and bone anabolic drugs. Herein, we described the case of a 4-year-old girl with GSD with extensive spinal involvement. She was treated with a combination of pamidronate, sirolimus, and atenolol. The patient had immediate clinical improvement after the administration of pamidronate. However, after 9 months of pamidronate and sirolimus treatment, the patient showed disease progression, and atenolol was added. After combination therapy with pamidronate, sirolimus, and atenolol for 11 months, the patient was clinically stable, with modest radiological improvement other than scoliosis.

Bisphosphonates are a class of drugs widely used to treat osteoporosis that inhibit osteoclast-mediated bone resorption. Pamidronate is a nitrogenous bisphosphonate that affects bone metabolism by blocking farnesyl phosphate synthase in the mevalonate pathway. This prevents the synthesis of two metabolites, farnesyl-diphosphate and geranylgeranyl diphosphate, which are required for the prenylation of small GTPases (such as Ras, Rho, and Rac) in osteoclasts. Prenylation of these small proteins is important for mediating protein–protein and protein–membrane interactions. As a result, the inhibition of prenylation by a nitrogenous bisphosphonate disrupts various cellular processes in osteoclasts, such as cytoskeletal arrangement, membrane ruffling, and vesicle trafficking [19,20]. Bisphosphonates have very high affinity for bone surfaces because they bind to hydroxyapatite, an essential inorganic mineral in the bone. This allows bisphosphonates selective targeting of bone minerals and effective inhibition of osteoclast-mediated bone resorption [21,22]. Furthermore, nitrogenous bisphosphonates, particularly pamidronate and zoledronic acid, have been reported to exert antiangiogenic effects by inducing persistent and significant decreases in serum vascular endothelial growth factor (VEGF) levels [23]. Owing to these bone-specific, anti-osteoclastic, and antiangiogenic properties, nitrogenous bisphosphonates are widely used for GSD monotherapy or in combination.

Bisphosphonates have been proven to be effective and safe in GSD in adults and the pediatric population [2,3,5,7,24,25,26]. Acute adverse effects of bisphosphonates include flu-like symptoms such as mild fever, malaise, and myalgia. Symptoms are usually self-limiting or resolve within hours to a few days with the use of antipyretics or analgesics [27]. Hypocalcemia rarely occurs after oral bisphosphonate treatment but may occur after intravenous injection due to failure to release calcium from the bone [28]. Bisphosphonate-induced hypocalcemia is usually mild and asymptomatic; however, symptoms such as muscle cramps, tetany, and prolonged corrected QT intervals should be monitored. Symptomatic hypocalcemia should be treated with intravenous calcium gluconate or oral calcium supplements. Based on several clinical observations, calcium and vitamin D status should be assessed before intravenous administration and corrected if needed [29,30]. Osteonecrosis of the jaws has been reported in adults treated with bisphosphonates but not in pediatric patients [31]. We administered intravenous pamidronate over 2 years to our patient, and she experienced no adverse effects except for a low-grade fever during the third course of treatment, which resolved spontaneously within hours.

Notably, our patient showed rapid improvement in back pain and general condition after the administration of pamidronate. This prompt clinical improvement has been documented in some case reports, including a 45-year-old woman who had rapid disappearance of local pain after pamidronate monotherapy [24] and a child who described immediate improvement of local pain after intravenous neridronate added to the previously prescribed alpha-2b interferon [2]. A systematic review analyzing the effect of bisphosphonates in treating bone pain related to various pathologies documented that 20 of 24 studies reported a positive effect of bisphosphonates in relieving pain [32]. However, the mechanism by which bisphosphonates reduce pain remains unclear. Osteoclasts contribute to bone pain in patients with cancer with bone metastases by destroying bone and secreting protons, thereby creating acidic microenvironment [33,34]. Acidic conditions act as stimuli for nociceptive nerves that innervate the bone, and these nociceptors activate intracellular signaling pathways and transcription factors in sensory neurons [34,35]. Therefore, inhibition of osteoclast activity by bisphosphonates may reduce acidification of the bone microenvironment, leading to alleviation of pain. Anti-inflammatory properties of bisphosphonates have been suggested as another possible analgesic mechanism of bisphosphonates [36]. The anti-inflammatory effects of bisphosphonates, particularly nitrogenous bisphosphonates, also appear to depend on the aforementioned inhibition of the prenylation of small GTPases. Modification loss of small GTPases, secondary to the inhibition of protein prenylation by bisphosphonates, appears to induce apoptosis not only in osteoclasts but also in macrophages and tumor cells [20]. Macrophages play a role in the release of inflammatory cytokines; consequently, macrophage apoptosis may lead to decreased production of inflammatory cytokines. In addition to macrophage and tumor cell apoptosis, the prevention of Ras prenylation has been suggested to be relevant to anti-inflammatory effects. Ras proteins activate several pathways, such as the mitogen-activated protein kinase cascade and the phosphatidylinositol 3-kinase (PI3K)-protein kinase B (Akt)-mTOR pathway, which are important for the development of inflammation [36]. We presume that the anti-inflammatory properties of nitrogenous bisphosphonates, along with the prevention of an acidic microenvironment in the affected bone, led to the rapid improvement of pain and sustained analgesic effects in our patient. Regarding its efficacy and safety, it was reasonable to continue long-standing bisphosphonate treatment in our patient, although some cases of spontaneous remission of GSD have also been reported [37,38].

Sirolimus is an immunosuppressant that directly inhibits mTOR, a serine/threonine kinase regulated by PI3K and Akt [8]. The PI3K-Akt-mTOR pathway stimulates cell growth, inhibits apoptosis, and increases VEGF expression, thereby promoting cell proliferation, angiogenesis, and lymphangiogenesis [7]. Sirolimus has recently been used for GSD with encouraging results owing to its antiproliferative and antiangiogenic activities [3,7,8,9,39]. A recent large study on the use of sirolimus in treating vascular anomalies showed overall successful results in 80.4% of patients, and six of seven patients with GSD had favorable responses [40]. The side effects of sirolimus include bone marrow suppression, mucositis or stomatitis, and hypertriglyceridemia. However, sirolimus is usually well tolerated, even in neonates [39,40]. Our patient also did not experience side effects other than two episodes of spontaneously resolved oral mucositis and mild thrombocytopenia, which did not require dose reduction. As mentioned above, nitrogenous bisphosphonates can induce tumor cell apoptosis through the alteration of Ras prenylation, which prevents PI3K-Akt-mTOR pathway activation. Therefore, the combination of nitrogenous bisphosphonates and sirolimus may potentiate the downregulation of the PI3K-Akt-mTOR cascade. This synergistic effect of the two treatments has been clinically demonstrated in patients with bone malignancies treated with zoledronate and everolimus [41]. An 18-year-old male with GSD was successfully treated with a combination of zoledronate and sirolimus [7]. However, in our case, although we similarly expected a synergistic effect of pamidronate and sirolimus, our patient showed an increased extent of paraspinal soft tissue lesions despite 9 months of combination therapy. We speculated that the low normal therapeutic range of sirolimus was a possible cause of her poor radiological response. We targeted a therapeutic range of 5–15 ng/mL of sirolimus, which is often considered sufficient to treat vascular anomalies [40]. Our patient usually had levels between 4 and 8 ng/mL; since younger children may require lower doses to achieve the therapeutic level, she continued on her dose of 1.6 mg/m^2^/day. We hypothesized that targeting a high normal therapeutic range may have led to a more favorable response. However, considering that the dose and duration of sirolimus in the treatment of vascular anomalies are still not established and whether sirolimus shows a dose-dependent effect in treating vascular anomalies requires further research, we are unable to conclusively determine the effect of low normal therapeutic targets on the treatment response in our patient.

Propranolol, a lipophilic nonselective beta-blocking agent, has been widely used to treat infantile hemangiomas because of its antiangiogenic activity. Beta blockers lead to vasoconstriction by decreasing nitric oxide release in the early phase, downregulating proangiogenic factors such as VEGF, basic fibroblast growth factor, and matrix metalloproteinases in the intermediate phase, and inducing capillary endothelium apoptosis in the late phase [42]. Ozeki et al. [43] reported impressive results for propranolol in intractable lymphangiomatosis, showing that propranolol inhibits lymphangiogenesis as well as angiogenesis. Since the first reported beneficial effect of propranolol in GSD [13], it has been used in a few patients with GSD [13,14,15]. Atenolol, a hydrophilic selective β_1_-blocker, was used in this case. To our knowledge, this is the first reported case of GSD treated with atenolol. We chose this drug based on recent studies that support the efficacy of atenolol over propranolol for infantile hemangiomas, with fewer adverse events, such as bradycardia, hypoglycemia, and bronchial reactivity, found with propranolol [16,17]. Atenolol does not affect pulmonary or pancreatic β_2_ receptors, which causes limited side effects compared to propranolol. Owing to its hydrophilic properties, it does not cross the blood–brain barrier, thus posing no risk of central nervous system side effects. Our patient had a decreased extent of paraspinal soft tissue lesions 4 months after atenolol was added to the combination therapy, and she did not experience any side effects from atenolol therapy.

Our case has several limitations. There was modest reduction in lesion size. However, we consider it clinically meaningful due to the prompt relief of back pain and the reduction in size shown shortly after the triple combination therapy. It should be noted that the patient previously showed radiographic progression, and after 4 months of triple combination therapy, the paraspinal lesion showed a decrease in size. Our rationale for the long-term bisphosphonate treatment derives from this immediate clinical improvement and stabilization of progressive osteolysis, while the extent of reduction was small. For further reduction in lesion size, zoledronic acid, the more potent nitrogenous bisphosphonate, is under consideration as the next therapeutic option for our patient. Pamidronate has been the most widely used and the most extensively reported agent in children with primary and secondary osteoporosis [44]. Zoledronic acid has been increasingly used in children, proving its efficacy and safety comparable or superior to the former use of pamidronate [45,46,47,48]. Zoledronic acid is considerably more potent than pamidronate in inhibiting osteoclast-mediated bone resorption and increasing trabecular bone mass [49]. Moreover, zoledronic acid has been reported to induce proliferation of osteoblasts in very low concentrations, while the proliferative effects on osteoblasts are lost in high concentrations [50]. Bisphosphonates bind to the bone tissue strongly, but a very low concentration of the drug is constantly released. Thus, the low-level zoledronic acid release may exert the proliferative effects on osteoblasts. In addition, recent evidence indicates that zoledronic acid directly interacts with ion channels such as the TRPV1 channel and ATP-Sensitive K^+^ channels [50]. In particular, zoledronic acid potentiates TRPV1 channels leading to mineralization of osteoblasts [51]. Therefore, although limited evidence is available concerning the use of zoledronic acid in young children with GSD, we presume this high-potent drug which also has anabolic activity via osteoblast proliferation and mineralization may bring a more favorable outcome to our patient. In addition, her progressed scoliosis still remains a challenge. It is difficult to determine the optimal timing of surgical spinal stabilization in GSD due to the progressive osteolysis and, in many cases, minimal bone stalk left for reconstruction. For successful surgical spinal stabilization, the most important factor is the disease status. If there is ongoing osteolysis, reconstruction failure and bone graft resorption is more likely. We are currently in discussion with the orthopedic surgeons regarding the timing of surgical intervention.

## 4. Conclusions

The triple combination treatment with bisphosphonates, sirolimus, and atenolol may have been helpful in stabilizing the disease course of GSD with early onset at 4 years of age. The best results were achieved in our patient after treatment with pamidronate, sirolimus, and atenolol, although treatment with sirolimus was not as successful as previously reported. Furthermore, bisphosphonates were effective in the immediate relief of pain as well as the prevention of bone resorption and were well tolerated. Atenolol showed promising results in this patient, supporting the therapeutic combination of an antiangiogenic agent and anti-osteoclast agent for the treatment of GSD. Long-term follow-up and further studies using bisphosphonates, sirolimus, and atenolol in GSD will help evaluate the efficacy of this therapeutic combination.

## Data Availability

The original contributions generated for the study are included in the article, further inquiries can be directed to the corresponding author.
